# Redefining Therapeutic Approaches in Colorectal Cancer: Targeting Molecular Pathways and Overcoming Resistance

**DOI:** 10.3390/ijms252312507

**Published:** 2024-11-21

**Authors:** Simona Gabriela Duta-Ion, Ioana Ruxandra Juganaru, Iulian Andrei Hotinceanu, Andra Dan, Livia Malina Burtavel, Madalin Codrut Coman, Ina Ofelia Focsa, Andra Giorgiana Zaruha, Patricia Christina Codreanu, Laurentiu Camil Bohiltea, Viorica Elena Radoi

**Affiliations:** 1 Department of Medical Genetics, “Carol Davila” University of Medicine and Pharmacy, 020021 Bucharest, Romania; simona-gabriela.duta@rez.umfcd.ro (S.G.D.-I.); iulian.hotinceanu@s.unibuc.ro (I.A.H.); andra.dan@rez.umfcd.ro (A.D.); livia-malina.burtavel@rez.umfcd.ro (L.M.B.); madalin-codrut.coman@rez.umfcd.ro (M.C.C.); ina.focsa@umfcd.ro (I.O.F.); andra-giorgiana.zaruha@rez.umfcd.ro (A.G.Z.); patricia-christina.codreanu@rez.umfcd.ro (P.C.C.); laurentiu.bohiltea@umfcd.ro (L.C.B.); viorica.radoi@umfcd.ro (V.E.R.); 2 “Alessandrescu-Rusescu” National Institute for Maternal and Child Health, 20382 Bucharest, Romania

**Keywords:** colon cancer, targeted therapy, immunotherapy, BRAF mutations, MSI/dMMR, PI3K/AKT/mTOR pathway, KRAS mutations, APC pathway

## Abstract

Colorectal cancer (CRC) arises through a combination of genetic and epigenetic alterations that affect key pathways involved in tumor growth and progression. This review examines the major molecular pathways driving CRC, including Chromosomal Instability (CIN), Microsatellite Instability (MSI), and the CpG Island Methylator Phenotype (CIMP). Key mutations in genes such as APC, KRAS, NRAS, BRAF, and TP53 activate signaling pathways like Wnt, EGFR, and PI3K/AKT, contributing to tumorigenesis and influencing responses to targeted therapies. Resistance mechanisms, including mutations that bypass drug action, remain challenging in CRC treatment. This review highlights the role of molecular profiling in guiding the use of targeted therapies such as tyrosine kinase inhibitors and immune checkpoint inhibitors. Novel combination treatments are also discussed as strategies to improve outcomes and overcome resistance. Understanding these molecular mechanisms is critical to advancing personalized treatment approaches in CRC and improving patient prognosis.

## 1. Introduction

Colorectal cancer (CRC) represents the third most common cause of death in Europe, representing 11.6% of all cancer deaths and directly taking approximately 215,000–250,000 human lives in the European population yearly [1]. In Romania, colorectal cancer is a significant health concern, ranking as the most common cancer across both sexes, with approximately 13,541 new cases recorded in 2022, representing 12.9% of all cancer cases in the country [2].

The general risk factors are well known in both the scientific community and the general population. Despite this, the incidence of most gastrointestinal malignancies is on the rise, especially in younger generations when compared to counterparts in older cohort studies, making the need for novel effective therapies imperative [3].

Targeted therapies have revolutionized colorectal cancer treatment in recent years, focusing on specific genes, proteins, or the tissue environment, which is crucial for cancer cell survival and growth. These therapies act through various mechanisms, either by blocking signals that tell cancer cells to grow and divide, like tyrosine kinase inhibitors (TKI), or by inducing cell death (apoptosis) in cancerous cells. Another targeted approach is preventing the formation of blood vessels (angiogenesis) that supply the tumor [4].

The first such agent approved by a regulatory body was cetuximab (marketed as Erbitux), approved by the U.S. Food and Drug Administration (FDA) in 2004 for use in metastatic colorectal cancer (mCRC). The second revolution came with the discovery of small molecule inhibitors, especially kinase inhibitors, which are that drugs interfere with specific enzymes or receptors within the cancer cells. The first tyrosine kinase inhibitor approved for colorectal cancer was regorafenib (marketed as Stivarga), which was approved by the U.S. FDA in 2012. The first non-tyrosine kinase small molecule inhibitor approved for colorectal cancer was trifluridine/tipiracil (marketed as Lonsurf), which was approved by the U.S. FDA in 2015 [4,5].

Since then, the therapeutic options have become broader, especially with the help of oncogenetics and molecular tumor profiling. The aim of this paper is to present an up-to-date review of current and upcoming molecular agents used for the treatment of both mCRC and CRC.

## 2. Key Molecular Pathways in Colorectal Cancer Carcinogenesis

**Figure 1:**
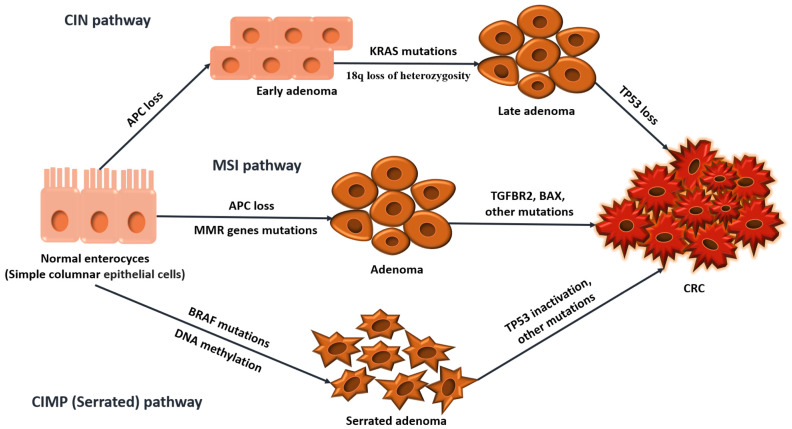
The three major molecular pathways in CRC carcinogenesis.

In colorectal cancer, three major molecular pathways drive carcinogenesis: the Chromosomal Instability (CIN) pathway, Microsatellite Instability (MSI) pathway, and the CpG Island Methylator Phenotype (CIMP) pathway [6]. Although these pathways provide a framework for understanding CRC’s genetic and molecular changes, tumors often do not follow a single pathway exclusively. Instead, significant overlap and interaction between these pathways exist, highlighting the complexity of CRC and the necessity for personalized treatments that target multiple pathways simultaneously. The main pathways of carcinogenesis are illustrated in Figure 1 below.

CIN is observed in over 70% of CRCs, typically involving mutations in the APC gene and affecting the distal colon. These tumors are characterized by chromosomal abnormalities, including mutations in KRAS and loss of TP53, and often exhibit loss of heterozygosity on chromosome 18, which contributes to tumor progression [7].

MSI is found in up to 30% of CRCs, particularly in tumors located in the proximal colon. This instability occurs when errors during DNA replication, particularly in microsatellite regions, go uncorrected due to defects in the DNA mismatch repair (MMR) system [8,9]. Key genes involved in this process include MLH1, MSH2, MSH6, and PMS2, which play an important role in correcting replication errors [8]. MSI is especially significant in Lynch syndrome, where inherited mutations in these MMR genes result in a higher risk of CRC [10]. MSI tumors frequently show mutations in TGFBR2, BAX and also APC, TCF7L2, and CTNNB1, activating the Wnt signaling pathway [7,11].

CIMP, also known as the Serrated Neoplasia Pathway, is implicated in around 15% of CRCs and is frequently associated with BRAF mutations and high Microsatellite Instability (MSI-H) status. These tumors are typically located in the proximal colon, are more prevalent in older females, and often arise from serrated polyps, characterized by a distinctive “saw-toothed” histological pattern [12]. The pathway is driven by epigenetic modifications, primarily the hypermethylation of CpG islands in promoter regions of specific genes. The BRAF V600E mutation plays a central role by activating the MAPK/ERK pathway, leading to uncontrolled cell proliferation. Furthermore, this mutation facilitates the formation of repressive complexes, such as the MAFG (Musculoaponeurotic Fibrosarcoma Oncogene Homolog G) corepressor complex, which methylates promoter regions of target genes. This hypermethylation and histone modification silences crucial genes involved in DNA repair, apoptosis, and other regulatory processes. One significant gene often silenced is MLH1, a critical component of the mismatch repair system. MLH1 silencing disrupts DNA repair mechanisms, resulting in Microsatellite Instability, a hallmark of CIMP-high (CIMP-H) tumors. However, about half of CIMP-positive CRCs do not exhibit MLH1 methylation or MSI, reflecting the heterogeneity of this pathway [13]. From a clinical perspective, CIMP-positive CRCs have shown variable responses to chemotherapy, with some studies indicating poor outcomes with 5-fluorouracil (5-FU) therapy, while others suggest potential benefits from irinotecan-based regimens [14].

Overall, the molecular landscape of CRC is fluid, with tumors often engaging more than one pathway, which necessitates complex and personalized treatment strategies.

### 2.1. Actionable Molecular Pathways

#### 2.1.1. EGFR Pathway

The epidermal growth factor receptor (EGFR) plays an important role in colorectal cancer by activating key signaling pathways, such as Ras-MAPK and PI3K-Akt, which drive tumor growth, angiogenesis, and survival [15]. EGFR is expressed in 60–80% of CRC cases and contributes to tumorigenesis by dysregulating the cell cycle and promoting survival factors [16]. The overexpression of EGFR is linked to poor prognosis, making it a vital therapeutic target. Anti-EGFR monoclonal antibodies, such as cetuximab and panitumumab, have proven effective in treating metastatic CRC, but only in patients with RAS wild-type tumors. Mutations in KRAS and NRAS lead to constant activation of downstream pathways, bypassing EGFR inhibition and causing primary resistance to these therapies [15,17]. Additional biomarkers, such as BRAF mutations, PIK3CA mutations and PTEN loss, also contribute to resistance. BRAF mutations, found in approximately 10% of mCRC cases, are particularly concerning as they predict a poor response to anti-EGFR treatment [17].

Despite these challenges, research into novel resistance mechanisms, such as changes in EGFR ligands (e.g., amphiregulin and epiregulin) and other receptors like HER2, is ongoing. These findings underscore the complexity of resistance in CRC and highlight the need for comprehensive molecular profiling in clinical practice to better select patients for targeted therapies [18]. In order to address these obstacles, combination therapies targeting multiple pathways are under investigation, alongside the development of new agents, such as allosteric inhibitors targeting specific RAS mutations. These advances hold promise for more personalized and effective treatment strategies for mCRC patients, addressing the dynamic and evolving landscape of resistance mechanisms [19].

**Figure 2:**
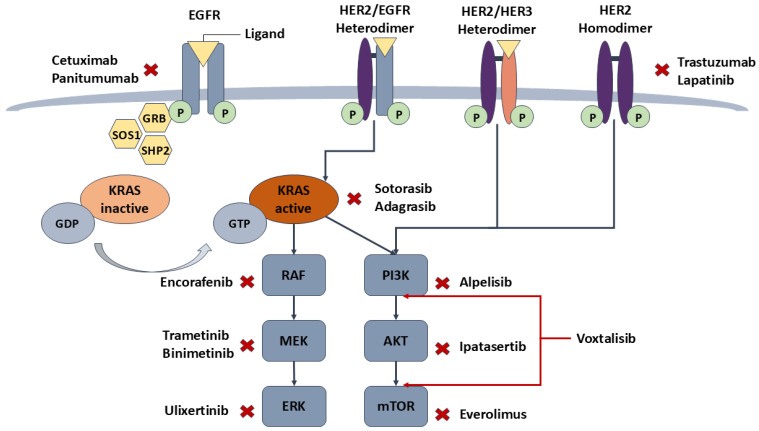
Inhibition points in the major molecular pathways and targeted treatment options.

Figure 2 below illustrates key therapeutic targets in the EGFR, KRAS, BRAF, and PI3K/AKT/mTOR pathways, with inhibitors that can block critical nodes in colorectal cancer progression.

#### 2.1.2. HER2 (ERBB2) Pathway

Human Epidermal Growth Factor Receptor 2 (HER2), also known as ERBB2 (Erythroblastic Oncogene B, Receptor Tyrosine Kinase 2), is a proto-oncogene that encodes a transmembrane glycoprotein receptor with tyrosine kinase activity. Unlike other members of the EGFR family, HER2 does not directly bind ligands. Instead, it is activated through homodimerization (pairing with another HER2 receptor) or heterodimerization (pairing with other receptors like EGFR, HER3, or HER4). These dimers trigger downstream signaling pathways involved in cell growth and survival, particularly the PI3K/AKT and MAPK/ERK pathways [20]. HER2 gene amplification or overexpression, observed in approximately 3–5% of metastatic colorectal cancers, is linked to aggressive disease behavior and poor response to anti-EGFR therapies. HER2 dimerization with HER3 is especially potent, leading to the strong activation of survival pathways, which complicates treatment [20,21].

Current guidelines recommend HER2 testing to identify patients who could benefit from targeted therapies, particularly those who are KRAS and BRAF wild-type. Testing methods like immunohistochemistry (IHC), in situ hybridization (ISH), and next-generation sequencing (NGS) are critical for accurate diagnosis and for guiding personalized treatment plans for HER2-positive patients [22,23].

In HER2-positive mCRC, first-line treatment generally involves chemotherapy combined with EGFR inhibitors or bevacizumab, depending on the RAS/BRAF mutation status [24]. Recent studies have highlighted the efficacy of HER2-targeted therapies, particularly in RAS/BRAF wild-type cases, with agents such as trastuzumab and tucatinib receiving accelerated FDA approval [25]. The HERACLES and MyPathway trials have further demonstrated the effectiveness of anti-HER2 combinations like trastuzumab with lapatinib or pertuzumab, underlining the importance of routine HER2 testing in mCRC patients [22,26,27].

Although HER2-targeted therapies have shown promise, overcoming treatment resistance remains a significant challenge. Ongoing research into combination strategies and molecular monitoring aims to optimize treatment outcomes for HER2-positive mCRC patients [20].

#### 2.1.3. KRAS Pathway

The Kirsten rat sarcoma viral oncogene homologue (KRAS) pathway plays a pivotal role in colorectal cancer oncogenesis, being involved in cell signaling processes that regulate proliferation, differentiation, and survival. KRAS, a member of the RAS family of oncogenes, encodes a GTPase protein that acts as a molecular switch in the RAS-RAF-MEK-ERK signaling pathway, which is responsible for transmitting signals from the cell surface to the nucleus, controlling gene expression and cellular behavior. KRAS gene mutations lead to the disruption of GTP hydrolysis, locking KRAS in its active form (GTP-bound state), resulting in persistent signaling and uncontrolled cell growth [28,29].

KRAS mutations are found in approximately 40% of CRC cases, with the majority occurring in codon 12 (around 65%) and the rest in codons 13 and 61 [29]. These mutations are frequently associated with right-sided colon tumors and with more aggressive disease phenotypes, including poor differentiation, advanced disease stage, and distant metastasis. Furthermore, KRAS mutations correlate with poorer survival and resistance to several therapeutic strategies [30].

The specific variants of KRAS mutations in CRC have distinct biological behaviors and clinical implications. Among the mutations at codon 12, the G12D (glycine to aspartic acid) and G12V (glycine to valine) variants are the most common and are associated with more aggressive disease and worse prognosis [31]. Notably, codon 13 mutations, specifically G13D, have a slightly better prognosis, and some studies suggest that CRC patients with G13D mutations may benefit from certain EGFR inhibitors, such as cetuximab [31,32,33].

KRAS mutations can also be used as predictive biomarkers for response to EGFR inhibitors therapy, which are widely used in the treatment of metastatic CRC. Cetuximab and panitumumab, monoclonal antibodies targeting EGFR, are only effective in patients with KRAS wild-type tumors [34,35]. Up to 50% of the patients harboring KRAS mutations are resistant to these therapies, highlighting the importance of genetic testing in clinical practice [32].

The development of KRAS G12C inhibitors represents a breakthrough in CRC targeted therapy. Sotorasib (AMG 510) and adagrasib (MRTX849) are small-molecule inhibitors specifically designed to target the G12C variant. They bind covalently to the cysteine residue, locking KRAS in its inactive GDP-bound state, thereby halting downstream signaling and tumorigenesis. Sotorasib has shown significant efficacy in preclinical models and early-phase clinical trials, particularly in NSCLC patients. However, its effectiveness in CRC has been less promising, probably due to the reactivation of EGFR signaling, which circumvents the inhibitory effects of sotorasib in CRC [36,37]. As a result, combination therapies, including KRAS G12C and EGFR inhibitors, are being explored to enhance therapeutic outcomes [38,39].

Despite these advances, targeting KRAS mutations directly remains challenging due to the high affinity of KRAS for GTP and the lack of suitable binding pockets for small-molecule inhibitors. Moreover, the indiscriminate inhibition of both wild-type and mutant KRAS may lead to significant toxicity [40]. To overcome these challenges, current research is focused on developing allosteric inhibitors and combination therapies that can selectively target mutant KRAS. This strategy holds promise for improving outcomes in patients with KRAS-mutant CRC [41].

#### 2.1.4. NRAS Pathway

Although less frequent than KRAS mutations, neuroblastoma ras viral oncogene homolog (NRAS) mutations occur in 5–9% of colorectal cancer cases, typically associated with tumor progression and resistance to anti-EGFR therapies in KRAS wild-type tumors [42]. NRAS and KRAS mutations share phenotypic traits, such as promoting tumorigenicity, but certain NRAS variants like Q61K can enhance tumor proliferation, while others like G12D might reduce proliferation and increase apoptosis. NRAS mutations at codons 12 and 61 activate pathways including IL1, JAK/STAT, and NF-κB, promoting an inflammatory tumor microenvironment, enhancing cell proliferation and survival, and contributing to therapy resistance [43]. Mutated NRAS loses its GTP hydrolysis ability, staying persistently active, which drives oncogenic signaling and complicates targeting due to its “undruggable” nature [44].

Furthermore, mutant NRAS protects colonic epithelial cells from stress-induced apoptosis, a function crucial in chronic inflammation contexts like inflammatory bowel disease. This anti-apoptotic role is mediated via the RAF-1 and STAT3 pathway, a unique mechanism distinguishing NRAS from KRAS or HRAS. The BEACON CRC trial highlights promising therapeutic avenues, as the combination of encorafenib (BRAF inhibitor), binimetinib (MEK inhibitor), and cetuximab (anti-EGFR antibody) has demonstrated a survival benefit (9.0 months) and a 26% response rate in advanced CRC, underscoring the efficacy of multi-targeted regimens in overcoming pathway reactivation [44,45]. Other strategies, especially those targeting the MAPK, PI3K, and RAL pathways, remain essential in addressing resistant CRC cases [43].

Moreover, a new structural study has identified a therapeutic target in NRAS Q61K’s switch II region, offering the potential for new inhibitors. This structural insight, along with targeting the NRAS-STAT3 axis, could significantly enhance treatment options for patients with NRAS-mutant CRC [44].

#### 2.1.5. BRAF Pathway

Mutations in the v-raf murine sarcoma viral oncogene homolog B1 (BRAF) gene, which encodes a serine/threonine protein kinase, also lead to the constitutive activation of RAS-RAF-MEK-ERK cascade, resulting in unchecked cell growth and tumor development. In CRC, approximately 8–10% of patients harbor BRAF mutations, with the BRAF V600E variant being the most common one [46]. This mutation results in a substitution of valine to glutamic acid at position 600, which mimics phosphorylation of the kinase activation loop, causing uncontrolled cell proliferation, independent of RAS activity [47]. The BRAF V600E mutation induces aggressive tumor biology and is associated with right-sided colon tumors, poor differentiation, and metastatic disease. Studies have shown that this mutation correlated with worse overall survival and progression-free survival compared to BRAF wild-type tumors [48]. Furthermore, these patients are less likely to benefit from standard chemotherapy regimens such as FOLFOX (5-fluorouracil, leucovorin, and oxaliplatin) and FOLFIRI (5-fluorouracil, leucovorin, and irinotecan) [49].

Early attempts at using BRAF inhibitors such as vemurafenib, which has been successful in treating BRAF-mutant melanoma, showed limited efficacy in CRC, mostly due to rapid reactivation of EGFR signaling, a compensatory mechanism that allows cancer cells to bypass BRAF inhibition [50]. This feedback activation of EGFR is particularly pronounced in CRC compared to other cancers. Given the limitations of BRAF inhibitors as monotherapy, combination therapies targeting multiple nodes within the signaling pathway have emerged as more effective strategies. Encorafenib, a small-molecule BRAF inhibitor, disrupts the MAPK/ERK signaling pathway, leading to decreased tumor cell proliferation and survival [51]. The BEACON trial evaluated the combination of encorafenib with cetuximab (an EGFR inhibitor), and in some cases, binimetinib (a MEK inhibitor), in patients with metastatic CRC with BRAF V600E mutation. The combination demonstrated significant improvement in survival rate, with an increase in median overall survival to 9.3 months, compared to 5.9 months for patients receiving standard chemotherapy [52].

MEK inhibitors, such as trametinib and binimetinib, target the MEK proteins, which are direct substrates of BRAF, thereby providing a more comprehensive blockade of the signaling pathway. However, the BEACON trial showed that adding binimetinib to encorafenib and cetuximab provided only marginal additional benefit over the two-drug combination [53].

Another combination regimen, using encorafenib, cetuximab, and an ERK1/2 selective inhibitor, ulixertinib, led to significant tumor regression in the BRAF V600E-mutant CRC model in preclinical studies. The regimen suggests great potential in overcoming resistance and enhancing treatment efficacy in comparison with monotherapy or double combination treatment [54].

Despite these advancements, BRAF V600E-mutant CRC remains difficult to treat. The resistance mechanisms that develop in response to combination therapy, particularly through the activation of alternate pathways such as the PI3K/AKT, present ongoing challenges. Preclinical models have suggested that further combination approaches may help to overcome resistance [55]. Immune checkpoint inhibitors, such as anti-PD1 antibodies, have shown limited efficacy as a monotherapy in microsatellite-stable BRAF-mutant CRC but may provide benefits when combined with targeted therapies or in tumors exhibiting high Microsatellite Instability [56,57].

#### 2.1.6. PI3K/mTOR Pathway

The PI3K/AKT/mTOR pathway plays an essential role in regulating a variety of cellular processes, including growth, metabolism, survival, and proliferation. In colorectal cancer, dysregulation of this pathway is a common event that contributes to oncogenesis. Central to this pathway is phosphoinositide 3-kinase (PI3K), a lipid kinase that phosphorylates PIP2 to produce PIP3, which then activates AKT (known as protein kinase B). Activated AKT phosphorylates and inactivates several downstream targets, including the mammalian target of rapamycin (mTOR), a key regulator of protein synthesis, cell growth, and metabolism. Mutations and amplifications in genes encoding components of this pathway, particularly PIK3CA, are frequently observed in CRC and play a significant role in tumorigenesis [58,59].

PIK3CA mutations are found in approximately 15–20% of CRC cases. These mutations lead to the constitutive activation of PI3K signaling, promoting uncontrolled cell proliferation. The most common ones are E542K, E545K, and H1047R mutations, which are frequently associated with resistance to various standard therapies, including EGFR inhibitors [60,61]. Additionally, alterations in other components of the pathway, such as PTEN loss (a tumor suppressor that negatively regulates PI3K signaling), are also seen in CRC and enhance oncogenic potential [62].

Despite the critical role of the PI3K/AKT/mTOR pathway in CRC, therapeutic targeting has proven challenging. Feedback loops and crosstalk with other pathways, such as RAS/RAF/MEK/ERK, can lead to compensatory mechanisms that undermine the efficacy of specific inhibitors. For instance, the inhibition of PI3K often leads to activation the of MAPK pathway, reducing the therapeutic impact of the targeted treatment [63]. However, several PI3K inhibitors are being investigated for their potential to overcome these challenges.

One of the most prominent PI3K inhibitors is alpelisib, a PI3Kα inhibitor that has shown efficacy in breast cancer [64]. Early-phase trials suggested that alpelisib may benefit patients with PIK3CA-mutant CRC, particularly when combined with other agents, such as anti-EGFR therapies or MEK inhibitors, although several more recent studies reported contradictory findings regarding the synergistic effect [64,65,66,67].

Ipatasertib (GDC-0068) is a potent, ATP-competitive, selective AKT inhibitor that targets all three AKT isoforms. Preclinical and clinical data shows its utility as a promising therapeutic option for CRC when used alone or in combination with standard therapies, especially in tumors with PTEN loss or PI3K pathway mutations [68,69].

Another therapeutic approach involves mTOR inhibitors, such as everolimus. Similarly to other targeted agents, mTOR inhibitors have been more effective when used in combination with PI3K inhibitors or cytotoxic chemotherapy. This combination strategy aims to block multiple points in the PI3K/AKT/mTOR pathway, reducing the likelihood of resistance [67].

To address the limitations of single-agent therapies, dual PI3K/mTOR inhibitors have been developed. Voxtalisib (XL765) is one such agent that has shown potential in preclinical models and early-phase clinical trials for CRC. Early studies suggest that dual inhibition may offer improved outcomes, though further investigation is necessary to optimize its clinical application [67,70].

#### 2.1.7. MSI/dMMR Pathway

The DNA mismatch repair (MMR) system is essential for maintaining genomic stability by identifying and correcting replication errors, such as short insertions, deletions, and base mismatches. Deficiencies in MMR proteins (dMMR) lead to Microsatellite Instability, a key biomarker in approximately 15% of CRC cases [71]. Tumors with dMMR/MSI-H status often have high mutation rates, resulting in more favorable outcomes in early-stage cancers but poorer prognoses in metastatic settings, particularly with mutations like BRAFV600E. This instability also reduces the effectiveness of standard chemotherapy [72].

Diagnostic and Treatment Guidelines recommend assessing MMR status in all CRC cases, irrespective of stage. Immunohistochemistry and polymerase chain reaction (PCR) are standard initial diagnostic tools with a high concordance rate (90–97%) [71]. While IHC is preferred as the first-line test, NGS has recently been approved by the FDA for MSI/dMMR testing, providing expanded analysis capabilities. New algorithms, such as MSICare, are also being developed to improve MSI detection sensitivity and specificity [71,73].

The introduction of Immune Checkpoint Inhibitors (ICIs), such as pembrolizumab and nivolumab, has transformed treatment for MSI-H/dMMR metastatic CRC. The KEYNOTE-177 trial established pembrolizumab as the standard first-line therapy for MSI-H/dMMR mCRC, showing significant improvements in progression-free survival (PFS) and overall survival (OS) compared to chemotherapy alone [74]. Additional trials, such as those investigating atezolizumab (a PD-L1 inhibitor) combined with bevacizumab (a VEGF inhibitor), have shown a 30% objective response rate (ORR) and a 90% disease control rate in MSI-H mCRC patients pretreated with chemotherapy. The combination of atezolizumab, bevacizumab, and FOLFOX chemotherapy achieved an ORR of 52% and a PFS of 14.1 months, regardless of MSI status [16,72].

Resistance mechanisms in dMMR/MSI-H CRC include the development of alternative immune escape mechanisms, such as loss of antigen presentation and T-cell exclusion within the tumor microenvironment. Mutations in RAS and BRAFV600E, as well as immune milieu alterations, have been associated with reduced response to PD-1 inhibitors [73]. Addressing these challenges, current research is exploring combination therapies, such as dual checkpoint blockade with nivolumab and ipilimumab, which have shown higher response rates in early clinical trials. The CheckMate-142 trial demonstrated promising outcomes, with increased response rates and survival in previously treated MSI-H/dMMR mCRC patients [75].

Integrating ICIs with targeted therapies, especially for BRAFV600E-mutant patients, has shown potential. For example, the SEAMARK trial is currently assessing pembrolizumab combined with encorafenib (a BRAF inhibitor) and cetuximab (an EGFR inhibitor) in untreated BRAFV600E-mutant MSI-H/dMMR mCRC [73]. These combination therapies aim to overcome limitations associated with standard ICIs by targeting specific genetic alterations alongside immune checkpoints.

**Figure 3:**
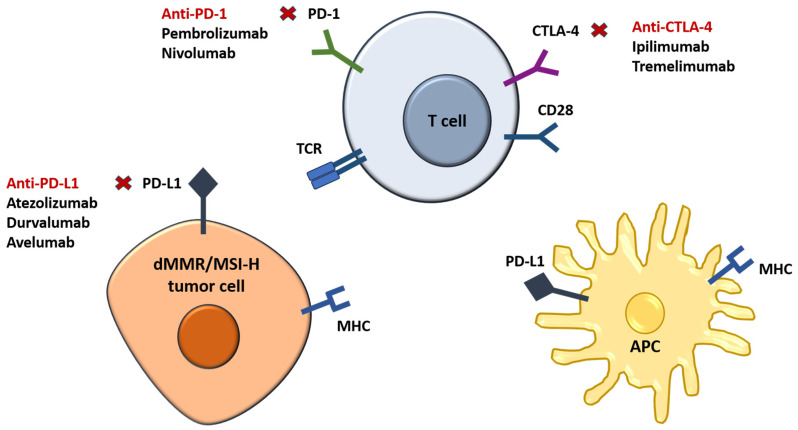
Mechanism of action of current checkpoint inhibitors in CRC.

Immune checkpoint inhibitors act by blocking inhibitory signals, enhancing the immune system’s ability to recognize and destroy dMMR/MSI-H colorectal cancer cells, and improving treatment outcomes in affected patients; the process is depicted in Figure 3.

#### 2.1.8. APC Pathway

Another oncogenic pathway in CRC is the Adenomatous polyposis coli (APC) pathway, particularly by regulating the Wnt/β-catenin signaling. APC is a tumor suppressor gene that encodes a protein responsible for β-catenin degradation, a key regulator of gene transcription that controls cell proliferation and differentiation [76,77].

APC gene mutations are found in about 80–90% of sporadic colorectal cancers, making it one of the most common and earliest genetic alterations in CRC tumorigenesis [78]. These mutations typically result in a truncated APC protein that loses its ability to form the β-catenin destruction complex, leading to the accumulation of β-catenin in the nucleus. Consequently, β-catenin drives the expression of Wnt target genes such as MYC, CCND1 (cyclin D1), and AXIN2, promoting uncontrolled cell division and, subsequently, the development of adenomas and carcinomas [79].

The loss of APC function is a hallmark of early colorectal adenoma formation, and studies have shown that this alteration frequently precedes other key mutations, such as those in KRAS or TP53, in the multistep model of colorectal carcinogenesis. Germline mutations in APC are also responsible for familial adenomatous polyposis (FAP), a hereditary condition characterized by the development of hundreds to thousands of colorectal adenomas and an almost inevitable progression to CRC if left untreated [80]. The importance of APC in regulating Wnt/β-catenin signaling highlights its critical role in both hereditary and sporadic forms of CRC.

Despite the central position of the APC pathway in CRC, directly targeting APC mutations or the Wnt/β-catenin pathway for therapeutic purposes has proven challenging. The Wnt/β-catenin pathway is highly conserved and is involved in numerous physiological processes, making it difficult to selectively target this pathway without causing significant off-target effects and toxicity. Additionally, β-catenin lacks an easily druggable binding site, further complicating the development of inhibitors against this protein [81].

However, indirect therapeutic strategies targeting downstream or regulatory components of the Wnt/β-catenin pathway are under investigation. One promising approach involves inhibiting tankyrase, an enzyme that regulates β-catenin stability through the degradation of axin, a key scaffold protein in the β-catenin destruction complex. Inhibiting tankyrase increases the levels of axin, thereby enhancing the degradation of β-catenin and reducing its oncogenic signaling [82]. Preclinical studies using tankyrase inhibitors, such as G007-LK, have demonstrated the potential to restore β-catenin degradation and inhibit tumor growth in APC-mutant CRC models [83]. Despite these promising results, tankyrase inhibitors are still in the early stages of clinical development, and their safety and efficacy in humans are yet to be fully established.

Another strategy focuses on PORCN inhibitors, which target Porcupine, an enzyme required for the secretion and activity of Wnt ligands. Through the inhibition of Wnt ligand secretion, PORCN inhibitors can effectively block Wnt signaling, reducing the nuclear accumulation of β-catenin and its oncogenic effects. LGK974, a PORCN inhibitor, has shown efficacy in preclinical CRC models and is currently undergoing early-phase clinical trials in patients with Wnt-driven cancers, including CRC [84].

Furthermore, other approaches are being explored to target Wnt/β-catenin signaling in CRC. These include small-molecule inhibitors targeting β-catenin interactions with other proteins, such as CBP/p300, which are essential for β-catenin-mediated transcription. By disrupting these interactions, it may be possible to reduce β-catenin’s oncogenic transcriptional activity. PRI-724 is one such compound that targets the CBP/β-catenin interaction and has shown potential in preclinical studies, and more recently, in early human trials, though its clinical efficacy remains under further investigation [85,86].

Immunotherapy is also being explored as a treatment strategy for CRC with dysregulated APC/Wnt/β-catenin signaling. Recent studies have shown that activation of the Wnt pathway in tumors can lead to an immunosuppressive tumor microenvironment, limiting the effectiveness of immune checkpoint inhibitors, such as anti-PD-1 antibodies. Therefore, combining Wnt pathway inhibitors with immunotherapy could enhance anti-tumor immune responses and improve outcomes for patients with APC-mutant CRC [87].

#### 2.1.9. TP53 Pathway

The TP53 pathway is also of great importance in the regulation of cell cycle control, apoptosis, and DNA repair, playing a central role in maintaining genomic integrity. The TP53 gene encodes the tumor suppressor protein p53, a transcription factor activated in response to various cellular stress signals such as DNA damage, hypoxia, and oncogene activation. Once activated, p53 can induce cell cycle arrest, DNA repair, senescence, or apoptosis, preventing the propagation of damaged cells that could otherwise develop into cancer [88,89].

In colorectal cancer, mutations in TP53 occur in approximately 50–60% of cases, particularly in the later stages of tumor progression. The majority of TP53 mutations are missense mutations that result in a loss of function or dominant-negative effects, allowing cancer cells to evade apoptosis and continue proliferating. Unlike other tumor suppressors, such as APC, which are usually inactivated early in CRC development, TP53 mutations are typically acquired later in the adenoma–carcinoma sequence, contributing to the transition from a benign adenoma to an invasive carcinoma [88,90].

The p53 protein functions primarily as a transcription factor, so the mutated form of TP53 loses its ability to regulate key genes involved in cell cycle control and apoptosis, such as CDKN1A (p21), BAX, and PUMA. Mutant p53 proteins allow the accumulation of various genetic alterations due to DNA damage and can also gain oncogenic properties by promoting cell migration, invasion, and metabolic reprogramming, further contributing to the malignancy of CRC [91,92].

Unlike oncogenes such as KRAS or BRAF, which can be inhibited by small molecules targeting their constitutive activation, restoring the function of mutant p53 or overcoming its loss of function has proven difficult from a pharmacological perspective. However, recent advances have led to the development of several promising therapeutic strategies that aim to either reactivate mutant p53 or exploit the vulnerabilities of p53-deficient cancers [89,91].

MDM2 inhibitors block the interaction between MDM2 and p53, preventing p53 degradation and enhancing its tumor-suppressive function. These inhibitors, like idasanutlin, are mainly effective in tumors with wild-type TP53, but may also benefit some TP53-mutant CRCs that still have functional p53 [93]. Early clinical trials of idasanutlin have shown promising results, especially when combined with chemotherapy or targeted treatments [94].

APR-246 (eprenetapopt) is a small-molecule that can restore p53 function, promoting its refolding and stabilizing its structure. Preclinical studies have demonstrated that APR-246 can induce apoptosis in TP53-mutant CRC cell lines, and early-phase clinical trials have shown activity in TP53-mutant hematologic malignancies [95].

Additionally, there is a growing interest in exploiting the synthetic lethality concept, with TP53-deficient tumors being more vulnerable to certain therapies due to their reliance on compensatory survival pathways. For instance, p53-deficient cancers are more dependent on CHK1 and ATR for cell cycle checkpoint control, sensitizing them to inhibitors of these kinases [96,97]. CHK1 inhibitors, such as prexasertib, and ATR inhibitors, such as ceralasertib, are being evaluated in clinical trials for their ability to selectively kill TP53-mutant or p53-deficient tumors by exploiting these vulnerabilities [98,99].

Immunotherapy is also emerging as a potential approach for treating TP53-mutant CRC. Mutant p53 proteins often generate neoantigens recognizable by the immune system, making these tumors potentially more responsive to immune checkpoint inhibitors, such as anti-PD-1/PD-L1 therapies. Recent studies have suggested that p53 mutations may correlate with an increased tumor mutational burden (TMB) and a more immunogenic tumor microenvironment, although the efficacy of immunotherapy in microsatellite-stable (MSS) CRCs with TP53 mutations is yet to be fully explored [100,101,102].

#### 2.1.10. NTRK Fusions

Neurotrophic tropomyosin kinase receptor (NTRK) fusions arise when the NTRK genes (NTRK1, NTRK2, and NTRK3) fuse with other unrelated genes, generating abnormal proteins that stimulate uncontrolled cancer growth. The TRK family of tyrosine kinases—TRKA, TRKB, and TRKC—encoded by these genes is crucial for regulating cell growth, differentiation, and survival. Typically, these receptors are activated by neurotrophins, triggering downstream pathways like MAPK, PI3K/AKT, and PLC-γ. When fused with other genes, however, the TRK receptors become permanently active, driving persistent cell proliferation and promoting cancer progression [18].

Although NTRK fusions are relatively uncommon, accounting for less than 1% of solid tumors, they occur more frequently in specific rare cancers, such as secretory carcinoma of the salivary gland and congenital infantile fibrosarcoma. In colorectal cancer, the most frequent fusion involves the TPM3-NTRK1 rearrangement. Detection methods for NTRK fusions include IHC, fluorescence in situ hybridization (FISH), and next-generation sequencing (NGS) [103,104]. The majority of NTRK alterations, however, are variants of unknown significance (VUS) and are often missense mutations, making them not currently actionable. These mutations are more prevalent in tumors with a high tumor mutation burden (TMB > 10), though their clinical relevance is still not fully understood [105]. Given the therapeutic potential, systematic screening for NTRK fusions in dMMR/MSI-H CRC patients, particularly those RAS/RAF wild-type, is recommended to optimize treatment strategies [106].

A study analyzing 7008 colonic adenocarcinomas revealed a 0.23% occurrence rate for NTRK fusions, primarily involving NTRK1 and NTRK3 [105]. Common fusion partners included TPM3, LMNA, TPR, and EML4, leading to the aberrant activation of TRK proteins, which stimulate oncogenic pathways such as MAPK and PI3K/AKT, resulting in unchecked cell proliferation. Clinically, patients with NTRK fusion-positive CRC were predominantly women, and their tumors were typically located in the right colon, exhibiting moderate to poor differentiation. Additionally, many of these tumors had a mucinous component and high levels of tumor-infiltrating lymphocytes, often associated with Microsatellite Instability [105,107].

The discovery of NTRK fusions has led to the development of targeted therapies, including larotrectinib and entrectinib, both TRK inhibitors designed to block the activity of these aberrant TRK proteins [108,109]. These first-generation NTRK tyrosine kinase inhibitors have been approved by the U.S. FDA for both adult and pediatric patients. The clinical benefits observed in NTRK fusion-positive cancers, regardless of tumor location or histology, highlight the importance of NTRK fusions as biomarkers for personalized cancer treatment, offering a pathway to targeted therapeutic interventions [18,107].

Table 1 summarizes the molecular pathways implicated in CRC carcinogenesis and the major therapeutic strategies previously discussed.

### 2.2. Future Directions in CRC Treatment

Chimeric Antigen Receptor (CAR)-T cell therapy, a groundbreaking immunotherapy, has revolutionized the treatment of certain hematological malignancies, demonstrating remarkable efficacy in conditions like leukemia and lymphoma. This success triggered interest in investigating CAR T-cell therapy applications for solid tumors, including colorectal cancer. CAR T-cell therapy involves engineering T cells to express chimeric antigen receptors (CARs) that recognize tumor-specific antigens (TSAs) or tumor-associated antigens (TAAs) [110]. However, translating the therapy to CRC presents various challenges, primarily due to the tumor microenvironment (TME), which is often immunosuppressive, hindering the infiltration and activity of CAR T-cells. The TME in CRC is characterized by hypoxia and suppressive immune components, such as regulatory T cells and myeloid-derived suppressor cells. Additionally, immune checkpoint molecules, particularly the PD-1/PD-L1 axis, inhibit T-cell activity, enabling tumors to evade immune detection. Furthermore, the identification of suitable antigens in CRC is complex, as many potential targets are also expressed in normal tissues, raising concerns about treatment toxicity [111,112].

TSAs, derived from tumor-specific mutations, are highly specific but rare in CRC. TAAs, such as epithelial cell adhesion molecule (EpCAM), carcinoembryonic antigen (CEA), and guanylyl cyclase C (GUCY2C), are more abundant, but pose risks of off-target effects due to expression in normal tissues [111,113].

Despite these limitations, ongoing research is exploring innovative strategies to enhance CAR T-cell efficacy against CRC. These include engineering CAR T-cells with improved tumor-penetrating capabilities, developing dual-targeting CARs to increase specificity, and combining CAR T-cell therapy with other treatments to modulate the tumor microenvironment [112].

Preclinical studies have shown significant promise. EpCAM-targeted CAR-T cells suppressed peritoneal metastases in mouse models, while CEA-specific CAR-T cells showed improved tumor infiltration and efficacy when combined with cytokines like IL-12 or when delivered intraperitoneally [113,114]. Similarly, GUCY2C-directed CAR-T cells demonstrated tumor suppression with minimal toxicity to healthy tissues, emphasizing their specificity [115].

CAR-T cells targeting stress-induced antigens, such as heat shock protein 70 (Hsp70) and placental alkaline phosphatase (PLAP), have shown specificity and reduced toxicity [116,117]. Combining CAR-T therapy with immune checkpoint inhibitors, such as anti-PD-1 or LAG-3 antibodies, has enhanced CAR-T cells persistence and restored cytotoxicity function, commonly suppressed by TME [113].

A promising advancement is CD6-CAR-T cells targeting CD166, a glycoprotein highly expressed in CRC but minimally in normal tissues. These cells demonstrated potent cytotoxicity against CRC cells and cancer stem cells, which play a central role in drug resistance, metastasis, and recurrence. CD6-CAR-T cells effectively suppressed epithelial–mesenchymal transition (EMT) markers, linked to tumor aggressiveness and CSC survival. Importantly, they spared non-tumor cells with low CD166 expression, highlighting their precision and safety [118].

Other innovative strategies complement CAR-T therapy. Bispecific antibodies (BiTEs), which connect T cells to tumor cells by targeting CD3 on T cells and TAAs like EpCAM or CEA, have shown efficacy against KRAS- and BRAF-mutated CRC cells, where conventional therapies fail. mRNA-lipid nanoparticle delivery systems have further enhanced BiTE specificity and efficacy [119]. While still in experimental stages, these advancements hold promise for integrating CAR T-cell therapy into the therapeutic arsenal against colorectal cancer.

Tumor-infiltrating lymphocytes (TILs) are another promising approach. TIL therapy involves expanding immune cells extracted from tumors ex vivo and reinfusing them into patients. While effective in cancers like melanoma, CRC poses challenges due to lower TIL abundance. Nevertheless, ongoing clinical trials are refining this therapy [119,120].

Oncolytic viruses (OVs) provide a unique mechanism by selectively infecting and destroying tumor cells while activating anti-tumor immunity. Adenoviruses, herpes simplex viruses (HSV), and reoviruses have shown potential in preclinical studies [121]. For example, the vaccinia virus JX-594 (Pexa-Vec) effectively treated peritoneal metastases by restoring peritoneal anti-tumor immunity [122].

Anti-tumor vaccines also hold promise by stimulating immune responses against TSAs or TAAs. Platforms include whole tumor vaccines, peptide antigen vaccines, viral vector vaccines, and dendritic cell (DC) vaccines [123]. While whole tumor vaccines have shown limited efficacy due to poor immunogenicity, peptide antigen vaccines targeting CEA or mucin-1 have demonstrated positive trends in clinical trials, albeit with HLA-restricted applicability [124]. Viral vector vaccines, such as the CEA/TRICOM platform, and DC vaccines, which use autologous DCs to activate T cells, are also under investigation, though challenges like cost and scalability remain [125].

Combining these novel therapies with traditional treatments, such as chemotherapy or immune checkpoint blockade, offers significant potential to overcome resistance and improve CRC outcomes. These advancements represent a shift toward personalized approaches in CRC treatment.

## 3. Conclusions

Genetic testing has become pivotal in diagnosing and treating CRC, enabling a tailored approach based on the tumor’s molecular profile. Personalized management is especially critical given CRC’s rising incidence among younger populations, notably those aged 40–50, which impacts both productivity and healthcare systems, particularly in low-resource regions with limited advanced medical access [4]. NGS and molecular profiling facilitate key gene mutation detection, such as KRAS, NRAS, BRAF, and PIK3CA. These mutations not only provide insight into tumor behavior, but also allow the choice of targeted therapies.

Targeted therapies, particularly those inhibiting angiogenesis like bevacizumab, have become fundamental in CRC treatment [126]. For BRAF V600E-mutated tumors, combining encorafenib with anti-EGFR agents such as cetuximab has shown promise, as this approach disrupts multiple signaling pathways that contribute to tumor growth and chemotherapy resistance [127]. Furthermore, immunotherapies such as pembrolizumab and nivolumab, which target PD-1 to enhance T-cell responses, are being explored in combination with CTLA4 inhibitors like ipilimumab. These combinations are particularly beneficial for MMR-deficient and MSI-H CRC cases, which tend to respond favorably to immune checkpoint blockade [128].

A broad range of FDA-approved therapies now address specific molecular targets within CRC. Anti-EGFR antibodies like cetuximab and panitumumab are effective in RAS wild-type tumors, while agents targeting HER2, such as trastuzumab, are undergoing evaluation in HER2-positive CRC. Approved immunotherapies like pembrolizumab and nivolumab serve MSI-H/dMMR patients well due to their propensity for immune checkpoint inhibition. Notably, the combination of encorafenib and cetuximab provides a critical option for patients with BRAF V600E-mutant CRC, a population previously limited to chemotherapy [52].

Despite these advances, therapeutic resistance remains a major hurdle, prompting research into combination therapies that counteract resistance pathways. The SUNLIGHT Phase 3 trial, for instance, explores the combination of trifluridine/tipiracil (FTD/TPI) with bevacizumab to extend survival in resistant CRC cases. Comparisons with established therapies have shown that FTD/TPI is well-tolerated, offering an option for patients unable to endure irinotecan’s side effects. Novel therapies like fruquintinib, a selective VEGFR inhibitor, show encouraging results in clinical trials, such as FRESCO-2, underscoring the evolving landscape of CRC treatments [4].

Future CRC strategies involve leveraging advanced nanocarriers, such as polymeric nanoparticles, to improve drug delivery to tumor cells while sparing healthy tissue. This approach aligns with the goal of personalized medicine by minimizing toxicity and optimizing drug efficacy [129]. Additionally, innovative immunotherapy combinations, as seen in the AtezoTRIBE study, combine immune checkpoint inhibitors with VEGF/VEGFR and anti-angiogenesis agents, which could significantly enhance outcomes in metastatic, hard-to-treat cases [130,131]. Ongoing trials underscore the potential of multi-modal therapy approaches to improve survival and reduce resistance in CRC, highlighting the promise of innovative treatment paradigms for CRC patients.

Current treatments integrating chemotherapy, immunotherapy, and targeted agents show improved outcomes, yet challenges remain with therapy resistance. Ongoing research into biomarkers, novel combinations, and adaptive strategies offers hope for enhanced efficacy and broader treatment options, paving the way for future advances in CRC care.

## Figures and Tables

**Table 1:** Main pathways, therapeutic strategies and treatment response in CRC.

Pathway	Prevalence	Therapeutic Response	Future Strategies
EGFR	Overexpressed in many CRCs	Anti-EGFR therapies (cetuximab, panitumumab) are effective in RAS wild-type tumors.	Combination therapies targeting EGFR and downstream pathways; developing allosteric inhibitors for specific RAS mutations.
HER2	~3–5% of mCRC cases	Anti-HER2 therapies (trastuzumab, lapatinib) are effective, particularly in HER2+ tumors.	Combining HER2-targeted therapies with agents like EGFR or BRAF inhibitors to overcome resistance.
KRAS	~40% of CRC cases	EGFR inhibitors (cetuximab, panitumumab) are only effective in KRAS wild-type tumors.	Development of KRAS G12C inhibitors (e.g., sotorasib) and combination therapies targeting multiple pathways.
NRAS	~5–9% of CRC cases	MEK/ERK inhibitors are explored due to constitutive activation of MAPK/PI3K pathways.	Developing inhibitors targeting newly discovered structural sites in NRAS mutants (e.g., Q61K); targeting the NRAS-STAT3 axis.
BRAF	~8–10% of CRC cases	Combination of BRAF (encorafenib), EGFR (cetuximab), and MEK inhibitors	Combination therapies targeting BRAF, EGFR, and MEK inhibitors; immune checkpoint inhibitors for MSI-H BRAF tumors.
PI3K/mTOR	~15–20% of CRC cases	PI3K inhibitors (alpelisib) and mTOR inhibitors (everolimus) show promise in combination therapies.	Dual PI3K/mTOR inhibitors and combination with anti-EGFR or MEK inhibitors to address resistance.
MSI/dMMR	~15% of CRC cases	Strong response to immune checkpoint inhibitors (e.g., pembrolizumab, nivolumab).	Combination therapies, dual checkpoint blockade (e.g., nivolumab + ipilimumab), or BRAF/EGFR inhibitors for BRAFV600E tumors.
APC	~80–90% of CRC cases	Targeting tankyrase (G007-LK) and PORCN inhibitors (LGK974) in preclinical trials.	Indirect strategies such as tankyrase or PORCN inhibitors; combination with immunotherapy to overcome immunosuppressive TME.
TP53	~50–60% of CRC cases	MDM2 inhibitors (idasanutlin) and mutant p53 reactivators (APR-246) show preclinical promise.	Exploiting synthetic lethality with CHK1/ATR inhibitors; immunotherapy for tumors with p53 neoantigens.
NTRK Fusions	<1% of CRC cases	TRK inhibitors (larotrectinib, entrectinib) are highly effective across cancers with NTRK fusions.	Early detection of NTRK fusions and combination therapies to prevent resistance.

## References

[1] Eurostat Cancer Statistics—Specific Cancers—Statistics Explained. https://ec.europa.eu/eurostat/statistics-explained/index.php?title=Cancer_statistics_-_specific_cancers#Colorectal_cancer.

[2] Bray F., Laversanne M., Sung H., Ferlay J., Siegel R.L., Soerjomataram I., Jemal A. (2024). Global Cancer Statistics 2022: GLOBOCAN Estimates of Incidence and Mortality Worldwide for 36 Cancers in 185 Countries. CA Cancer J. Clin..

[3] Sifaki-Pistolla D., Poimenaki V., Fotopoulou I., Saloustros E., Mavroudis D., Vamvakas L., Lionis C. (2022). Significant Rise of Colorectal Cancer Incidence in Younger Adults and Strong Determinants: 30 Years Longitudinal Differences between under and over 50 s. Cancers.

[4] Xie Y.-H., Chen Y.-X., Fang J.-Y. (2020). Comprehensive Review of Targeted Therapy for Colorectal Cancer. Signal Transduct. Target. Ther..

[5] Kumar A., Gautam V., Sandhu A., Rawat K., Sharma A., Saha L. (2023). Current and Emerging Therapeutic Approaches for Colorectal Cancer: A Comprehensive Review. World J. Gastrointest. Surg..

[6] Pierantoni C., Cosentino L., Ricciardiello L. (2024). Molecular Pathways of Colorectal Cancer Development: Mechanisms of Action and Evolution of Main Systemic Therapy Compunds. Dig. Dis..

[7] Farooqi A.A., de la Roche M., Djamgoz M.B.A., Siddik Z.H. (2019). Overview of the Oncogenic Signaling Pathways in Colorectal Cancer: Mechanistic Insights. Semin. Cancer Biol..

[8] Battaglin F., Naseem M., Lenz H.-J., Salem M.E. (2018). Microsatellite Instability in Colorectal Cancer: Overview of Its Clinical Significance and Novel Perspectives. Clin. Adv. Hematol. Oncol..

[9] Pećina-Šlaus N., Kafka A., Salamon I., Bukovac A. (2020). Mismatch Repair Pathway, Genome Stability and Cancer. Front. Mol. Biosci..

[10] Zeinalian M., Hashemzadeh-Chaleshtori M., Salehi R., Emami M.H. (2018). Clinical Aspects of Microsatellite Instability Testing in Colorectal Cancer. Adv. Biomed. Res..

[11] Testa U., Pelosi E., Castelli G. (2018). Colorectal Cancer: Genetic Abnormalities, Tumor Progression, Tumor Heterogeneity, Clonal Evolution and Tumor-Initiating Cells. Med. Sci..

[12] Rhee Y.-Y., Kim K.-J., Kang G.H. (2017). CpG Island Methylator Phenotype-High Colorectal Cancers and Their Prognostic Implications and Relationships with the Serrated Neoplasia Pathway. Gut Liver.

[13] Nguyen L.H., Goel A., Chung D.C. (2020). Pathways of Colorectal Carcinogenesis. Gastroenterology.

[14] Zhang X., Zhang W., Cao P. (2021). Advances in CpG Island Methylator Phenotype Colorectal Cancer Therapies. Front. Oncol..

[15] Tardito S., Matis S., Zocchi M.R., Benelli R., Poggi A. (2024). Epidermal Growth Factor Receptor Targeting in Colorectal Carcinoma: Antibodies and Patient-Derived Organoids as a Smart Model to Study Therapy Resistance. Int. J. Mol. Sci..

[16] Pabla B., Bissonnette M., Konda V.J. (2015). Colon Cancer and the Epidermal Growth Factor Receptor: Current Treatment Paradigms, the Importance of Diet, and the Role of Chemoprevention. World J. Clin. Oncol..

[17] Lu X., Li Y., Li Y., Zhang X., Shi J., Feng H., Yu Z., Gao Y. (2023). Prognostic and Predictive Biomarkers for Anti-EGFR Monoclonal Antibody Therapy in RAS Wild-Type Metastatic Colorectal Cancer: A Systematic Review and Meta-Analysis. BMC Cancer.

[18] Martini G., Ciardiello D., Vitiello P.P., Napolitano S., Cardone C., Cuomo A., Troiani T., Ciardiello F., Martinelli E. (2020). Resistance to Anti-Epidermal Growth Factor Receptor in Metastatic Colorectal Cancer: What Does Still Need to Be Addressed?. Cancer Treat. Rev..

[19] Zhao B., Wang L., Qiu H., Zhang M., Sun L., Peng P., Yu Q., Yuan X. (2017). Mechanisms of Resistance to Anti-EGFR Therapy in Colorectal Cancer. Oncotarget.

[20] Ahcene Djaballah S., Daniel F., Milani A., Ricagno G., Lonardi S. (2022). HER2 in Colorectal Cancer: The Long and Winding Road from Negative Predictive Factor to Positive Actionable Target. Am. Soc. Clin. Oncol. Educ. Book Am. Soc. Clin. Oncol. Meet..

[21] Sartore-Bianchi A., Amatu A., Porcu L., Ghezzi S., Lonardi S., Leone F., Bergamo F., Fenocchio E., Martinelli E., Borelli B. (2019). HER2 Positivity Predicts Unresponsiveness to EGFR-Targeted Treatment in Metastatic Colorectal Cancer. Oncologist.

[22] Roy-Chowdhuri S., Davies K.D., Ritterhouse L.L., Snow A.N. (2022). ERBB2 (HER2) Alterations in Colorectal Cancer. J. Mol. Diagn..

[23] Babkoff A., Zick A., Hubert A., Tarantino P., Grinshpun A. (2024). Unleashing the Power of Anti-HER2 Therapies in Metastatic Colorectal Cancer: Paving the Way for a Brighter Future. ESMO Gastrointest. Oncol..

[24] Robinson H.R., Messersmith W.A., Lentz R.W. (2024). HER2-Positive Metastatic Colorectal Cancer. Curr. Treat. Options Oncol..

[25] Zheng-Lin B., Bekaii-Saab T.S. (2024). Treatment Options for HER2-Expressing Colorectal Cancer: Updates and Recent Approvals. Ther. Adv. Med. Oncol..

[26] Tosi F., Sartore-Bianchi A., Lonardi S., Amatu A., Leone F., Ghezzi S., Martino C., Bencardino K., Bonazzina E., Bergamo F. (2020). Long-Term Clinical Outcome of Trastuzumab and Lapatinib for HER2-Positive Metastatic Colorectal Cancer. Clin. Colorectal Cancer.

[27] Meric-Bernstam F., Hurwitz H., Raghav K.P.S., McWilliams R.R., Fakih M., VanderWalde A., Swanton C., Kurzrock R., Burris H., Sweeney C. (2019). Pertuzumab plus Trastuzumab for *HER2*-Amplified Metastatic Colorectal Cancer (MyPathway): An Updated Report from a Multicentre, Open-Label, Phase 2a, Multiple Basket Study. Lancet Oncol..

[28] Network C.G.A. (2012). Comprehensive Molecular Characterization of Human Colon and Rectal Cancer. Nature.

[29] Prior I.A., Hood F.E., Hartley J.L. (2020). The Frequency of Ras Mutations in Cancer. Cancer Res..

[30] Allievi N., Goffredo P., Utria A.F., Pisano M., Poiasina E., Lucianetti A., Zhou P., Hassan I. (2019). The Association of KRAS Mutation with Primary Tumor Location and Survival in Patients Undergoing Resection of Colorectal Cancers and Synchronous Liver Metastases. Chin. Clin. Oncol..

[31] Yaeger R., Chatila W.K., Lipsyc M.D., Hechtman J.F., Cercek A., Sanchez-Vega F., Jayakumaran G., Middha S., Zehir A., Donoghue M.T.A. (2018). Clinical Sequencing Defines the Genomic Landscape of Metastatic Colorectal Cancer. Cancer Cell.

[32] Waring P., Tie J., Maru D., Karapetis C.S. (2016). RAS Mutations as Predictive Biomarkers in Clinical Management of Metastatic Colorectal Cancer. Clin. Colorectal Cancer.

[33] Loupakis F., Ruzzo A., Cremolini C., Vincenzi B., Salvatore L., Santini D., Masi G., Stasi I., Canestrari E., Rulli E. (2009). KRAS Codon 61, 146 and BRAF Mutations Predict Resistance to Cetuximab plus Irinotecan in KRAS Codon 12 and 13 Wild-Type Metastatic Colorectal Cancer. Br. J. Cancer.

[34] Lo Nigro C., Ricci V., Vivenza D., Granetto C., Fabozzi T., Miraglio E., Merlano M.C. (2016). Prognostic and Predictive Biomarkers in Metastatic Colorectal Cancer Anti-EGFR Therapy. World J. Gastroenterol. WJG.

[35] Amado R.G., Wolf M., Peeters M., Van Cutsem E., Siena S., Freeman D.J., Juan T., Sikorski R., Suggs S., Radinsky R. (2008). Wild-Type KRAS Is Required for Panitumumab Efficacy in Patients with Metastatic Colorectal Cancer. J. Clin. Oncol..

[36] Skoulidis F., Li B.T., Dy G.K., Price T.J., Falchook G.S., Wolf J., Italiano A., Schuler M., Borghaei H., Barlesi F. (2021). Sotorasib for Lung Cancers with KRAS p.G12C Mutation. N. Engl. J. Med..

[37] Fakih M.G., Kopetz S., Kuboki Y., Kim T.W., Munster P.N., Krauss J.C., Falchook G.S., Han S.-W., Heinemann V., Muro K. (2022). Sotorasib for Previously Treated Colorectal Cancers with KRASG12C Mutation (CodeBreaK100): A Prespecified Analysis of a Single-Arm, Phase 2 Trial. Lancet Oncol..

[38] Akhave N.S., Biter A.B., Hong D.S. (2021). Mechanisms of Resistance to KRASG12C-Targeted Therapy. Cancer Discov..

[39] Kasi P.M., Afable M.G., Herting C., Lukanowski M., Jin Z. (2023). Anti-EGFR Antibodies in the Management of Advanced Colorectal Cancer. Oncologist.

[40] McCormick F. (2015). KRAS as a Therapeutic Target. Clin. Cancer Res..

[41] Zhao D., Liu Y., Yi F., Zhao X., Lu K. (2023). Recent Advances in the Development of Inhibitors Targeting KRAS-G12C and Its Related Pathways. Eur. J. Med. Chem..

[42] Kuhn N., Klinger B., Uhlitz F., Sieber A., Rivera M., Klotz-Noack K., Fichtner I., Hoffmann J., Blüthgen N., Falk C. (2021). Mutation-Specific Effects of NRAS Oncogenes in Colorectal Cancer Cells. Adv. Biol. Regul..

[43] Loree J.M., Wang Y., Syed M.A., Sorokin A.V., Coker O., Xiu J., Weinberg B.A., Vanderwalde A.M., Tesfaye A., Raymond V.M. (2021). Clinical and Functional Characterization of Atypical KRAS/NRAS Mutations in Metastatic Colorectal Cancer. Clin. Cancer Res..

[44] Gebregiworgis T., Chan J.Y.-L., Kuntz D.A., Privé G.G., Marshall C.B., Ikura M. (2024). Crystal Structure of NRAS Q61K with a Ligand-Induced Pocket near Switch II. Eur. J. Cell Biol..

[45] Lin K.X., Istl A.C., Quan D., Skaro A., Tang E., Zheng X. (2023). PD-1 and PD-L1 Inhibitors in Cold Colorectal Cancer: Challenges and Strategies. Cancer Immunol. Immunother..

[46] O’Riordan E., Bennett M.W., Daly L., Power D.G. (2022). The Implication of BRAF Mutation in Advanced Colorectal Cancer. Ir. J. Med. Sci..

[47] Maloney R.C., Zhang M., Jang H., Nussinov R. (2021). The Mechanism of Activation of Monomeric B-Raf V600E. Comput. Struct. Biotechnol. J..

[48] Nakayama I., Hirota T., Shinozaki E. (2020). BRAF Mutation in Colorectal Cancers: From Prognostic Marker to Targetable Mutation. Cancers.

[49] Ciombor K.K., Strickler J.H., Bekaii-Saab T.S., Yaeger R. (2022). BRAF-Mutated Advanced Colorectal Cancer: A Rapidly Changing Therapeutic Landscape. J. Clin. Oncol..

[50] Corcoran R.B. (2015). New Therapeutic Strategies for BRAF Mutant Colorectal Cancers. J. Gastrointest. Oncol..

[51] Al-Salama Z.T. (2021). Encorafenib: A Review in Metastatic Colorectal Cancer with a BRAF V600E Mutation. Drugs.

[52] Scott K., Axel G., Rona Y., Eric V.C., Jayesh D., Takayuki Y., Harpreet W., Fortunato C., Fotios L., Sang H.Y. (2019). Encorafenib, Binimetinib, and Cetuximab in BRAF V600E–Mutated Colorectal Cancer. N. Engl. J. Med..

[53] Tabernero J., Grothey A., Van Cutsem E., Yaeger R., Wasan H., Yoshino T., Desai J., Ciardiello F., Loupakis F., Hong Y.S. (2021). Encorafenib plus Cetuximab as a New Standard of Care for Previously Treated BRAF V600E-Mutant Metastatic Colorectal Cancer: Updated Survival Results and Subgroup Analyses from the BEACON Study. J. Clin. Oncol..

[54] Sullivan R.J., Infante J.R., Janku F., Wong D.J.L., Sosman J.A., Keedy V., Patel M.R., Shapiro G.I., Mier J.W., Tolcher A.W. (2018). First-in-Class ERK1/2 Inhibitor Ulixertinib (BVD-523) in Patients with MAPK Mutant Advanced Solid Tumors: Results of a Phase I Dose-Escalation and Expansion Study. Cancer Discov..

[55] Ahronian L.G., Sennott E.M., Van Allen E.M., Wagle N., Kwak E.L., Faris J.E., Godfrey J.T., Nishimura K., Lynch K.D., Mermel C.H. (2015). Clinical Acquired Resistance to RAF Inhibitor Combinations in BRAF-Mutant Colorectal Cancer through MAPK Pathway Alterations. Cancer Discov..

[56] Zhong J., Sun Z., Li S., Yang L., Cao Y., Bao J. (2023). Immune Checkpoint Blockade Therapy for BRAF Mutant Metastatic Colorectal Cancer: The Efficacy, New Strategies, and Potential Biomarkers. Discover. Oncol..

[57] Le D.T., Uram J.N., Wang H., Bartlett B.R., Kemberling H., Eyring A.D., Skora A.D., Luber B.S., Azad N.S., Laheru D. (2024). PD-1 Blockade in Tumors with Mismatch-Repair Deficiency. N. Engl. J. Med..

[58] Fruman D.A., Chiu H., Hopkins B.D., Bagrodia S., Cantley L.C., Abraham R.T. (2017). The PI3K Pathway in Human Disease. Cell.

[59] Maharati A., Moghbeli M. (2023). PI3K/AKT Signaling Pathway as a Critical Regulator of Epithelial-Mesenchymal Transition in Colorectal Tumor Cells. Cell Commun. Signal..

[60] Cathomas G. (2014). PIK3CA in Colorectal Cancer. Front. Oncol..

[61] Voutsadakis I.A. (2021). The Landscape of PIK3CA Mutations in Colorectal Cancer. Clin. Colorectal Cancer.

[62] Ugai T., Zhao M., Shimizu T., Akimoto N., Shi S., Takashima Y., Zhong R., Lau M.C., Haruki K., Arima K. (2021). Association of PIK3CA Mutation and PTEN Loss with Expression of CD274 (PD-L1) in Colorectal Carcinoma. Oncoimmunology.

[63] Thorpe L.M., Yuzugullu H., Zhao J.J. (2015). PI3K in Cancer: Divergent Roles of Isoforms, Modes of Activation and Therapeutic Targeting. Nat. Rev. Cancer.

[64] André F., Ciruelos E., Rubovszky G., Campone M., Loibl S., Rugo H.S., Iwata H., Conte P., Mayer I.A., Kaufman B. (2019). Alpelisib for PIK3CA-Mutated, Hormone Receptor-Positive Advanced Breast Cancer. N. Engl. J. Med..

[65] Ye Y., Huang Z., Zhang M., Li J., Zhang Y., Lou C. (2023). Synergistic Therapeutic Potential of Alpelisib in Cancers (Excluding Breast Cancer): Preclinical and Clinical Evidences. Biomed. Pharmacother..

[66] Clarke P.A., Roe T., Swabey K., Hobbs S.M., McAndrew C., Tomlin K., Westwood I., Burke R., van Montfort R., Workman P. (2019). Dissecting Mechanisms of Resistance to Targeted Drug Combination Therapy in Human Colorectal Cancer. Oncogene.

[67] Leiphrakpam P.D., Are C. (2024). PI3K/Akt/MTOR Signaling Pathway as a Target for Colorectal Cancer Treatment. Int. J. Mol. Sci..

[68] Ferrari A., Merks J.H.M., Chisholm J.C., Orbach D., Brennan B., Gallego S., van Noesel M.M., McHugh K., van Rijn R.R., Gaze M.N. (2020). Outcomes of Metastatic Non-Rhabdomyosarcoma Soft Tissue Sarcomas (NRSTS) Treated within the BERNIE Study: A Randomised, Phase II Study Evaluating the Addition of Bevacizumab to Chemotherapy. Eur. J. Cancer.

[69] Isakoff S.J., Tabernero J., Molife L.R., Soria J.-C., Cervantes A., Vogelzang N.J., Patel M.R., Hussain M., Baron A., Argilés G. (2020). Antitumor Activity of Ipatasertib Combined with Chemotherapy: Results from a Phase Ib Study in Solid Tumors. Ann. Oncol..

[70] Schram A.M., Gandhi L., Mita M.M., Damstrup L., Campana F., Hidalgo M., Grande E., Hyman D.M., Heist R.S. (2018). A Phase Ib Dose-Escalation and Expansion Study of the Oral MEK Inhibitor Pimasertib and PI3K/MTOR Inhibitor Voxtalisib in Patients with Advanced Solid Tumours. Br. J. Cancer.

[71] Parente P., Grillo F., Vanoli A., Macciomei M.C., Ambrosio M.R., Scibetta N., Filippi E., Cataldo I., Baron L., Ingravallo G. (2023). The Day-to-Day Practice of MMR and MSI Assessment in Colorectal Adenocarcinoma: What We Know and What We Still Need to Explore. Dig. Dis..

[72] Mulet-Margalef N., Linares J., Badia-Ramentol J., Jimeno M., Sanz Monte C., Manzano Mozo J.L., Calon A. (2023). Challenges and Therapeutic Opportunities in the DMMR/MSI-H Colorectal Cancer Landscape. Cancers.

[73] Cervantes B., André T., Cohen R. (2024). Deficient Mismatch Repair/Microsatellite Unstable Colorectal Cancer: Therapeutic Advances and Questions. Ther. Adv. Med. Oncol..

[74] André T., Shiu K.-K., Kim T.W., Jensen B.V., Jensen L.H., Punt C., Smith D., Garcia-Carbonero R., Benavides M., Gibbs P. (2020). Pembrolizumab in Microsatellite-Instability-High Advanced Colorectal Cancer. N. Engl. J. Med..

[75] Lenz H.-J., Van Cutsem E., Luisa Limon M., Wong K.Y.M., Hendlisz A., Aglietta M., García-Alfonso P., Neyns B., Luppi G., Cardin D.B. (2021). First-Line Nivolumab Plus Low-Dose Ipilimumab for Microsatellite Instability-High/Mismatch Repair-Deficient Metastatic Colorectal Cancer: The Phase II CheckMate 142 Study. J. Clin. Oncol..

[76] Stamos J.L., Weis W.I. (2013). The β-Catenin Destruction Complex. Cold Spring Harb. Perspect. Biol..

[77] Clevers H., Nusse R. (2012). Wnt/β-Catenin Signaling and Disease. Cell.

[78] Christie M., Jorissen R.N., Mouradov D., Sakthianandeswaren A., Li S., Day F., Tsui C., Lipton L., Desai J., Jones I.T. (2013). Different APC Genotypes in Proximal and Distal Sporadic Colorectal Cancers Suggest Distinct WNT/β-Catenin Signalling Thresholds for Tumourigenesis. Oncogene.

[79] Valenta T., Hausmann G., Basler K. (2012). The Many Faces and Functions of β-Catenin. EMBO J..

[80] Leoz M.L., Carballal S., Moreira L., Ocaña T., Balaguer F. (2015). The Genetic Basis of Familial Adenomatous Polyposis and Its Implications for Clinical Practice and Risk Management. Appl. Clin. Genet..

[81] Zhan T., Rindtorff N., Boutros M. (2017). Wnt Signaling in Cancer. Oncogene.

[82] Lau T., Chan E., Callow M., Waaler J., Boggs J., Blake R.A., Magnuson S., Sambrone A., Schutten M., Firestein R. (2013). A Novel Tankyrase Small-Molecule Inhibitor Suppresses APC Mutation-Driven Colorectal Tumor Growth. Cancer Res..

[83] Arqués O., Chicote I., Puig I., Tenbaum S.P., Argilés G., Dienstmann R., Fernández N., Caratù G., Matito J., Silberschmidt D. (2016). Tankyrase Inhibition Blocks Wnt/β-Catenin Pathway and Reverts Resistance to PI3K and AKT Inhibitors in the Treatment of Colorectal Cancer. Clin. Cancer Res..

[84] Shah K., Panchal S., Patel B. (2021). Porcupine Inhibitors: Novel and Emerging Anti-Cancer Therapeutics Targeting the Wnt Signaling Pathway. Pharmacol. Res. Off. J. Ital. Pharmacol. Soc..

[85] Schmidtova S., Kalavska K., Liskova V., Plava J., Miklikova S., Kucerova L., Matuskova M., Rojikova L., Cierna Z., Rogozea A. (2021). Targeting of Deregulated Wnt/β-Catenin Signaling by PRI-724 and LGK974 Inhibitors in Germ Cell Tumor Cell Lines. Int. J. Mol. Sci..

[86] El-Khoueiry A.B., Ning Y., Yang D., Cole S., Kahn M., Zoghbi M., Berg J., Fujimori M., Inada T., Kouji H. (2024). A Phase I First-in-Human Study of PRI-724 in Patients (Pts) with Advanced Solid Tumors. J. Clin. Oncol..

[87] Luke J.J., Bao R., Sweis R.F., Spranger S., Gajewski T.F. (2019). WNT/β-Catenin Pathway Activation Correlates with Immune Exclusion across Human Cancers. Clin. Cancer Res..

[88] Hernández Borrero L.J., El-Deiry W.S. (2021). Tumor Suppressor P53: Biology, Signaling Pathways, and Therapeutic Targeting. Biochim. Biophys. Acta Rev. Cancer.

[89] Wang H., Guo M., Wei H., Chen Y. (2023). Targeting P53 Pathways: Mechanisms, Structures and Advances in Therapy. Signal Transduct. Target. Ther..

[90] Markowitz S.D., Bertagnolli M.M. (2009). Molecular Origins of Cancer: Molecular Basis of Colorectal Cancer. N. Engl. J. Med..

[91] Güllülü Ö., Hehlgans S., Rödel C., Fokas E., Rödel F. (2021). Tumor Suppressor Protein P53 and Inhibitor of Apoptosis Proteins in Colorectal Cancer-A Promising Signaling Network for Therapeutic Interventions. Cancers.

[92] Wade M., Li Y.-C., Wahl G.M. (2013). MDM2, MDMX and P53 in Oncogenesis and Cancer Therapy. Nat. Rev. Cancer.

[93] Shangary S., Wang S. (2008). Targeting the MDM2-P53 Interaction for Cancer Therapy. Clin. Cancer Res..

[94] Italiano A., Miller Jr W.H., Blay J.-Y., Gietema J.A., Bang Y.-J., Mileshkin L.R., Hirte H.W., Higgins B., Blotner S., Nichols G.L. (2021). Phase I Study of Daily and Weekly Regimens of the Orally Administered MDM2 Antagonist Idasanutlin in Patients with Advanced Tumors. Invest. New Drugs.

[95] Lehmann S., Bykov V.J.N., Ali D., Andrén O., Cherif H., Tidefelt U., Uggla B., Yachnin J., Juliusson G., Moshfegh A. (2012). Targeting P53 in Vivo: A First-in-Human Study with P53-Targeting Compound APR-246 in Refractory Hematologic Malignancies and Prostate Cancer. J. Clin. Oncol..

[96] Reinhardt H.C., Aslanian A.S., Lees J.A., Yaffe M.B. (2007). P53-Deficient Cells Rely on ATM- and ATR-Mediated Checkpoint Signaling through the P38MAPK/MK2 Pathway for Survival after DNA Damage. Cancer Cell.

[97] Kantidze O.L., Velichko A.K., Luzhin A.V., Petrova N.V., Razin S. (2018). V Synthetically Lethal Interactions of ATM, ATR, and DNA-PKcs. Trends Cancer.

[98] Giudice E., Huang T.-T., Nair J.R., Zurcher G., McCoy A., Nousome D., Radke M.R., Swisher E.M., Lipkowitz S., Ibanez K. (2024). The CHK1 Inhibitor Prexasertib in BRCA Wild-Type Platinum-Resistant Recurrent High-Grade Serous Ovarian Carcinoma: A Phase 2 Trial. Nat. Commun..

[99] Yap T.A., Krebs M.G., Postel-Vinay S., El-Khouiery A., Soria J.-C., Lopez J., Berges A., Cheung S.Y.A., Irurzun-Arana I., Goldwin A. (2021). Ceralasertib (AZD6738), an Oral ATR Kinase Inhibitor, in Combination with Carboplatin in Patients with Advanced Solid Tumors: A Phase I Study. Clin. Cancer Res..

[100] Carlsen L., Zhang S., Tian X., De La Cruz A., George A., Arnoff T.E., El-Deiry W.S. (2023). The Role of P53 in Anti-Tumor Immunity and Response to Immunotherapy. Front. Mol. Biosci..

[101] Sobhani N., D’Angelo A., Wang X., Young K.H., Generali D., Li Y. (2020). Mutant P53 as an Antigen in Cancer Immunotherapy. Int. J. Mol. Sci..

[102] Joshi R.S., Boichard A., Adashek J.J., Kurzrock R. (2022). High Tumor Amplification Burden Is Associated with TP53 Mutations in the Pan-Cancer Setting. Cancer Biol. Ther..

[103] Manea C.A., Badiu D.C., Ploscaru I.C., Zgura A., Bacinschi X., Smarandache C.G., Serban D., Popescu C.G., Grigorean V.T., Botnarciuc V. (2022). A Review of NTRK Fusions in Cancer. Ann. Med. Surg..

[104] Karan C., Tan E., Sarfraz H., Walko C.M., Kim R.D., Knepper T.C., Sahin I.H. (2024). Characterization of NTRK Alterations in Metastatic Colorectal Cancer. J. Clin. Oncol..

[105] Lasota J., Chłopek M., Lamoureux J., Christiansen J., Kowalik A., Wasąg B., Felisiak-Gołąbek A., Agaimy A., Biernat W., Canzonieri V. (2020). Colonic Adenocarcinomas Harboring NTRK Fusion Genes: A Clinicopathologic and Molecular Genetic Study of 16 Cases and Review of the Literature. Am. J. Surg. Pathol..

[106] Wu S., Liu Y., Shi X., Zhou W., Zeng X. (2023). Elaboration of NTRK-Rearranged Colorectal Cancer: Integration of Immunoreactivity Pattern, Cytogenetic Identity, and Rearrangement Variant. Dig. Liver Dis..

[107] O’Haire S., Franchini F., Kang Y.-J., Steinberg J., Canfell K., Desai J., Fox S., IJzerman M. (2023). Systematic Review of NTRK 1/2/3 Fusion Prevalence Pan-Cancer and across Solid Tumours. Sci. Rep..

[108] Kasi P.M., Afghan M.K., Bellizzi A.M., Chan C.H. (2022). Larotrectinib in Mismatch-Repair-Deficient TRK Fusion-Positive Metastatic Colon Cancer after Progression on Immunotherapy. Cureus.

[109] Doebele R.C., Drilon A., Paz-Ares L., Siena S., Shaw A.T., Farago A.F., Blakely C.M., Seto T., Cho B.C., Tosi D. (2020). Entrectinib in Patients with Advanced or Metastatic NTRK Fusion-Positive Solid Tumours: Integrated Analysis of Three Phase 1-2 Trials. Lancet Oncol..

[110] Li H., Yang C., Cheng H., Huang S., Zheng Y. (2021). CAR-T Cells for Colorectal Cancer: Target-Selection and Strategies for Improved Activity and Safety. J. Cancer.

[111] Qin X., Wu F., Chen C., Li Q. (2022). Recent Advances in CAR-T Cells Therapy for Colorectal Cancer. Front. Immunol..

[112] National Cancer Institute CAR T Cells: Engineering Patients’ Immune Cells to Treat Their Cancers. https://www.cancer.gov/about-cancer/treatment/research/car-t-cells.

[113] Ghazi B., El Ghanmi A., Kandoussi S., Ghouzlani A., Badou A. (2022). CAR T-Cells for Colorectal Cancer Immunotherapy: Ready to Go?. Front. Immunol..

[114] Wang L., Ma N., Okamoto S., Amaishi Y., Sato E., Seo N., Mineno J., Takesako K., Kato T., Shiku H. (2016). Efficient Tumor Regression by Adoptively Transferred CEA-Specific CAR-T Cells Associated with Symptoms of Mild Cytokine Release Syndrome. Oncoimmunology.

[115] Magee M.S., Kraft C.L., Abraham T.S., Baybutt T.R., Marszalowicz G.P., Li P., Waldman S.A., Snook A.E. (2016). GUCY2C-Directed CAR-T Cells Oppose Colorectal Cancer Metastases without Autoimmunity. Oncoimmunology.

[116] Bashiri Dezfouli A., Yazdi M., Benmebarek M.-R., Schwab M., Michaelides S., Miccichè A., Geerts D., Stangl S., Klapproth S., Wagner E. (2022). CAR T Cells Targeting Membrane-Bound Hsp70 on Tumor Cells Mimic Hsp70-Primed NK Cells. Front. Immunol..

[117] Li X., Berahovich R., Zhou H., Liu X., Li F., Xu S., Wei Y., Ouaret D., Bodmer W., Wu L. (2020). PLAP -CAR T Cells Mediate High Specific Cytotoxicity against Colon Cancer Cells. Front. Biosci. (Landmark Ed.).

[118] He S., Li S., Guo J., Zeng X., Liang D., Zhu Y., Li Y., Yang D., Zhao X. (2023). CD166-Specific CAR-T Cells Potently Target Colorectal Cancer Cells. Transl. Oncol..

[119] Kamrani A., Nasiri H., Hassanzadeh A., Ahmadian Heris J., Mohammadinasab R., Sadeghvand S., Sadeghi M., Valedkarimi Z., Hosseinzadeh R., Shomali N. (2024). New Immunotherapy Approaches for Colorectal Cancer: Focusing on CAR-T Cell, BiTE, and Oncolytic Viruses. Cell Commun. Signal..

[120] Bai Z., Zhou Y., Ye Z., Xiong J., Lan H., Wang F. (2021). Tumor-Infiltrating Lymphocytes in Colorectal Cancer: The Fundamental Indication and Application on Immunotherapy. Front. Immunol..

[121] Zolaly M.A., Mahallawi W., Khawaji Z.Y., Alahmadi M.A. (2023). The Clinical Advances of Oncolytic Viruses in Cancer Immunotherapy. Cureus.

[122] Ren Y., Miao J.-M., Wang Y.-Y., Fan Z., Kong X.-B., Yang L., Cheng G. (2022). Oncolytic Viruses Combined with Immune Checkpoint Therapy for Colorectal Cancer Is a Promising Treatment Option. Front. Immunol..

[123] Verma C., Pawar V.A., Srivastava S., Tyagi A., Kaushik G., Shukla S.K., Kumar V. (2023). Cancer Vaccines in the Immunotherapy Era: Promise and Potential. Vaccines.

[124] Martinis E., Ricci C., Trevisan C., Tomadini G., Tonon S. (2023). Cancer Vaccines: From the State of the Art to the Most Promising Frontiers in the Treatment of Colorectal Cancer. Pharmaceutics.

[125] Nikolouzakis T.K., Chrysos E., Docea A.O., Fragkiadaki P., Souglakos J., Tsiaoussis J., Tsatsakis A. (2024). Current and Future Trends of Colorectal Cancer Treatment: Exploring Advances in Immunotherapy. Cancers.

[126] Dinu I.M., Mihăilă M., Diculescu M.M., Croitoru V.M., Turcu-Stiolica A., Bogdan D., Miron M.I., Lungulescu C.V., Alexandrescu S.T., Dumitrașcu T. (2023). Bevacizumab Treatment for Metastatic Colorectal Cancer in Real-World Clinical Practice. Medicina.

[127] Antoniotti C., Rossini D., Pietrantonio F., Catteau A., Salvatore L., Lonardi S., Boquet I., Tamberi S., Marmorino F., Moretto R. (2022). Upfront FOLFOXIRI plus Bevacizumab with or without Atezolizumab in the Treatment of Patients with Metastatic Colorectal Cancer (AtezoTRIBE): A Multicentre, Open-Label, Randomised, Controlled, Phase 2 Trial. Lancet Oncol..

[128] Ganesh K., Stadler Z.K., Cercek A., Mendelsohn R.B., Shia J., Segal N.H., Diaz Jr L.A. (2019). Immunotherapy in Colorectal Cancer: Rationale, Challenges and Potential. Nat. Rev. Gastroenterol. Hepatol..

[129] C de S L Oliveira A.L., Schomann T., de Geus-Oei L.-F., Kapiteijn E., Cruz L.J., de Araújo Junior R.F. (2021). Nanocarriers as a Tool for the Treatment of Colorectal Cancer. Pharmaceutics.

[130] Adebayo A.S., Agbaje K., Adesina S.K., Olajubutu O. (2023). Colorectal Cancer: Disease Process, Current Treatment Options, and Future Perspectives. Pharmaceutics.

[131] Antoniotti C., Rossini D., Pietrantonio F., Salvatore L., Lonardi S., Tamberi S., Marmorino F., Moretto R., Prisciandaro M., Tamburini E. (2024). Upfront Fluorouracil, Leucovorin, Oxaliplatin, and Irinotecan Plus Bevacizumab With or Without Atezolizumab for Patients With Metastatic Colorectal Cancer: Updated and Overall Survival Results of the ATEZOTRIBE Study. J. Clin. Oncol..

